# Managing Binge Eating Disorder in a Young Adolescent Female: Barriers to Treatment and Recommendations

**DOI:** 10.7759/cureus.54237

**Published:** 2024-02-15

**Authors:** Rajesh K Mehta, Rahul Tyagi

**Affiliations:** 1 Child and Adolescent Psychiatry, Case Western Reserve University, MetroHealth Medical Center, Cleveland, USA; 2 Family Medicine, Leeds General Practitioner Confederation, Leeds, GBR; 3 Family Medicine, Royal College of General Practitioners, London, GBR

**Keywords:** access to health care, child and adolescent, family-based therapy, bupropion, binge eating disorder (bed)

## Abstract

Binge eating disorder (BED) is a behavioral disorder characterized by chronic and compulsive overeating. It is the most prevalent eating disorder in the USA, affecting almost 3% of the US population. In this study, we describe a case of BED in an adolescent Caucasian female who could not obtain specialized treatment because of financial constraints and saw a child and adolescent psychiatrist for medication management. Her treatment plan combined bupropion with family therapy and resulted in successful alleviation of her symptoms, allowing her to achieve a better quality of life. This case shows how pragmatism by PCPs can help manage patients who cannot obtain specialized care for their BED.

## Introduction

Binge eating disorder (BED) is characterized by eating a large quantity of food compared to what most people would within a given period, feeling a sense of compulsion or a lack of control associated with eating, and binge eating episodes occurring at least once a week that have been going on for several months. The other defining features of BED include binge eating occurring on average at least once a week for three months, marked distress regarding binge eating being present, and binge eating not being associated with the recurrent use of inappropriate compensatory behavior as in bulimia nervosa and not occurring exclusively during bulimia nervosa or anorexia nervosa [1]. Long-term consequences of BED include hypertension, cardiovascular disease, lipid abnormalities, type 2 diabetes, anxiety, and depression [1].

BED is the most common eating disorder (ED) in the United States, affecting people of all racial and ethnic groups. Around 1.25% of adult women and 0.42% of adult men have BED, with a lifetime prevalence ranging from 0.85% to 2.8% [1]. About 1.6% of teens aged 13 to 18 are affected [2]. The average age at which BED first manifests is 25 years. Almost two-thirds of people who meet the criteria for BED experience binge eating episodes over one year or longer [3,4].

Here, we describe a unique case where a 17-year-old female was experiencing a recurring episode of BED but could not access care at a specialized ED clinic because of financial constraints. This case shows the pivotal role that PCPs can potentially play in managing BED, especially given the scarcity of specialized ED clinics [5]. Combining psychotherapy and pharmacotherapy, PCPs can also help manage ED and improve quality of life.

## Case presentation

A 17-year-old Caucasian female living in Ohio, USA, was seen at an outpatient general child and adolescent clinic and was referred by her PCP for symptoms of BED, anxiety, and depression. Her symptoms had started during the pandemic, and during this time, she sought one session of cognitive behavioral therapy (CBT), leading to a brief episode of reduction in the binge eating episodes to once per week. Isolation during the COVID-19 pandemic was the initial trigger of her symptoms. The therapy was provided by her PCP’s integrated psychology team at the PCP; however, the patient did not find that this was helping because of its unstructured nature. After this period, there was a decline in her mental health with increased binging of up to five episodes per week, a decline in her personal care, with a weight gain of 25 pounds over two months. This was triggered by the stress of transitioning from high school to college. Moreover, she failed to attend sports practice and social activities. She would lose control and binge on any food items in the house. She was increasingly depressed with low mood and anhedonia symptoms.

She tried to maintain a healthy lifestyle by going to the gym daily. There was no history of suicidal or self-harm ideation. She lived with her parents and her sister. She had normal developmental milestones and no previous medical history. There was no family history of psychiatric disorders. She presented to the child and adolescent outpatient psychiatry clinic because her medical insurance did not cover treatment at a specialized ED center, and she would have to pay a substantial amount out of her pocket.

She was formally diagnosed with BED, anxiety, and depression by the psychiatrist. Both the patient and her guardian were open to medication management. The healthcare team provided extensive education to the patient's family regarding the diagnosis and treatment options (various pharmacological agents and therapy) for BED. A detailed review of her medical history showed no history of seizure disorders. Her basic laboratory results, including vitamin D, liver function test, complete blood count with differentials and basic metabolic profile, and thyroid function test, were within normal ranges. The Generalized Anxiety Disorder-7 (GAD-7) scale and Patient-Reported Outcomes Measurement Information System (PROMIS) were used. On initial assessment, her GAD-7 was 14, and PROMIS was 28.

During the discussions, the option of bupropion as a potential treatment was introduced after ruling out any seizure disorder. The patient demonstrated a positive response to this medication. The two-week review showed a clear improvement in her mood, anxiety, and binge eating episodes, which had decreased from four to five per week to one per week.

bupropion was initiated at 75 mg once a day and was gradually increased over eight weeks to a total dose of 300 mg once a day in the extended-release form. An additional 75 mg dose was added six months later in the evening for her anxiety and low mood. Family therapy was also incorporated into the treatment plan to address the psychological and emotional aspects of the disorder on the family and patient, helping reduce stress and improve family dynamics and coping skills. On subsequent reviews, the patient was doing very well. She was functioning well at school, at home, and with friends. She was having three meals a day with no symptoms of binge eating or restrictive behavior. Moreover, she noted mild weight loss and did not report any side effects. During her most recent review, she was optimistic about her future and attending college. There was marked improvement in her GAD-7 score from 14 initially to 7 recently and her PROMIS score from 28 initially to 32 recently.

## Discussion

This case study underscores the role that PCPs can play in managing the symptoms of BED and comorbidities when care in specialized settings is not accessible. As in this case, bupropion was prescribed by the child and adolescent psychiatrist for BED with anxiety and depression. The patient tolerated the medication well and had a positive response, which is an approach that PCPs can also take, initiating treatment sooner, rather than later. The psychiatry team will be available to assist and guide management if needed.

The primary barrier to treatment was the lack of insurance coverage for specialized ED center assessment and review, prompting a search for alternative approaches. With timely treatment, the patient experienced a noticeable reduction in binge eating episodes along with improved mood, improving her overall quality of life and mental health.

As with many mental health conditions, treatment of EDs can be difficult because of multiple factors such as gender perceptions, social/cultural barriers, stigma, and financial barriers.

Literature shows that females reported more symptoms associated with loss of control and distress because of overeating compared to males [6]. The gender disparity of symptoms and willingness to seek help for ED is postulated by the gender schema theory, in that symptom presentation patterns tend to conform to gender-normative schemas [7]. Males may be less likely to report eating-related distress or loss of control because expressing emotions may be more socially accepted and expected from females but often discouraged in males. Men's physiological differences and muscle mass may also be related to the differences seen in men presenting with few distress symptoms associated with overeating [6]. The cultural representation of ED, which is mostly more visible in women, may also impact the discrepancy between men and women. Men with BED symptoms are less likely to seek help as they may consider ED as a problem affecting women [6].

Ethnicity and race disparities also exist in seeking help for BED. Studies have shown that black women in the USA were significantly less likely to seek help and obtain treatment for BED [8]. Even in the UK, ethnic minorities are less likely to seek help in the UK compared to their white counterparts [9]. Sociocultural barriers and perceptions, such as ED impacting mainly white women, were reflected in the experience of women of Black, Asian, and Hispanic backgrounds not seeking help. The creation of such stigma within certain ethnically diverse societies was demonstrated by a qualitative study of 289 patients in 2010 [8].

Stigmatization of ED is also a hurdle in the way of seeking help for them. Often, patients may see an ED as a character flaw and think that healthcare professionals will not take them seriously or prioritize their management [8]. Hence, it is imperative to normalize the conversation not just around topics such as depression and anxiety but also other mental health conditions such as ED. 

Affordability of care is one of the main obstacles that patients must navigate to obtain help with BED and other EDs, especially in the USA, because of its unique health insurance structure. The Affordable Care Act has tried to address this, but many still fall through the cracks [10]. A retrospective chart review study of 1060 young patients (aged 11-25) with an ED showed the differences in care received depending on the insurance coverage. Compared to patients on private insurance, those on public insurance were up to 33% less likely to receive appropriate treatment [11]. Interestingly, another disparity between different ethnicities in the same study highlighted that Hispanic and Asian patients were up to 50% less likely to receive appropriate treatment compared to their white counterparts [11].

A lack of specialist physicians and centers within the desired traveling distance further compounds the issue of accessibility of cost-effective BED/ED management [12]. Project Heal, an ED organization in the USA, states that there are 228 ED treatment centers in the USA, with an average of 25 patients per program, and there are close to 6,000 available treatment spots in the USA. That equates to one spot for every 5,000 patients diagnosed with an ED, highlighting the lack of specialist centers and trained professionals to deal with ED [5].

Recommended treatment options for BED primarily include psychotherapy (CBT or interpersonal psychotherapy), with or without the addition of pharmacological agents, or some patients may wish to use medication only. Psychotherapy, particularly CBT, has proven to be more effective than the use of antidepressants alone for BED. However, the addition of antidepressant medication can help with reducing binge eating episodes irrespective of whether or not the individual has depressive symptoms [13]. There is no clear guidance on which antidepressant to use, but this generally depends on the side effect profile and any patient contraindications. Generally, tricyclic antidepressants and monoamine oxidase inhibitors are not recommended because of their adverse side effect profile and possible drug interactions [13].

Bupropion, an antidepressant of the aminoketone class, can be used either alone or in combination with naltrexone. It is a relatively safe drug to use, but a contraindication is a history of seizures. Purging activity is also contraindicated for bupropion use [13]. A double-blinded randomized controlled trial (RCT) study from 2013 demonstrated the use of bupropion alone in obese BED patients provided marked weight loss; however, there was no significant difference in reducing binge eating episodes or depressive symptoms compared to the placebo [14]. A recent double-blinded RCT compared the naltrexone-bupropion combination used alone or with behavioral weight loss therapy (BWL) [15]. Results showed that binge eating remission rates (defined as zero binge episodes in the previous four weeks) were 17.7% for the placebo group, 31.3% for the naltrexone-bupropion group, 37.1% for the BWL + placebo group, and 57.1% for the BWL + naltrexone-bupropion group. The study concluded that BWL + naltrexone-bupropion was superior to the rest of the groups in providing weight loss in obese subjects and helping patients achieve remission from binge eating episodes [15]. This observation provided justifiable clinical grounds to start bupropion and psychological therapy for this patient.

The only FDA-approved treatment for moderate to severe BED is lisdexamfetamine. It has shown improved weight loss in obese patients and has kept patients in remission from binge eating. Similar to other stimulants, there are side effects and contraindications where lisdexamfetamine should be used cautiously (e.g., people at risk of abuse of medication, heart disease, uncontrolled hypertension, history of psychosis, etc.) [13]. Topiramate, an anticonvulsant, is also recommended as a possible treatment choice for patients with BED and obesity. However, it has many side effects, including cognitive dysfunction. It is also teratogenic and is therefore avoided in young women of childbearing age [13].

## Conclusions

This case study demonstrates the importance of flexibility in treating BED when access to specialized centers is limited because of factors such as insurance coverage. Combining education, medication management, and, in selective cases, initiation by PCP in collaboration with a psychiatrist, and psychotherapy can be an effective way of managing BED.

Normalizing EDs through education is crucial so that people of all genders and ethnicities seek appropriate care for them when in need. Removing the stigma around EDs is also important for encouraging people to seek treatment for them. Additionally, concerted efforts are needed to make specialized care for EDs more accessible.
